# Prevention and Control of Seasonal Influenza with Vaccines: Recommendations of the Advisory Committee on Immunization Practices — United States, 2017–18 Influenza Season

**DOI:** 10.15585/mmwr.rr6602a1

**Published:** 2017-08-25

**Authors:** Lisa A. Grohskopf, Leslie Z. Sokolow, Karen R. Broder, Emmanuel B. Walter, Joseph S. Bresee, Alicia M. Fry, Daniel B. Jernigan

**Affiliations:** 1 Influenza Division, National Center for Immunization and Respiratory Diseases, CDC; 2 Battelle Memorial Institute, Atlanta, Georgia; 3 Immunization Safety Office, National Center for Emerging and Zoonotic Infectious Diseases, CDC; 4 Duke University School of Medicine, Durham, North Carolina

## Abstract

This report updates the 2016–17 recommendations of the Advisory Committee on Immunization Practices (ACIP) regarding the use of seasonal influenza vaccines (MMWR Recomm Rep 2016;65[No. RR-5]). Routine annual influenza vaccination is recommended for all persons aged ≥6 months who do not have contraindications. A licensed, recommended, and age-appropriate vaccine should be used.

For the 2017–18 season, quadrivalent and trivalent influenza vaccines will be available. Inactivated influenza vaccines (IIVs) will be available in trivalent (IIV3) and quadrivalent (IIV4) formulations. Recombinant influenza vaccine (RIV) will be available in trivalent (RIV3) and quadrivalent (RIV4) formulations. Live attenuated influenza vaccine (LAIV4) is not recommended for use during the 2017–18 season due to concerns about its effectiveness against (H1N1)pdm09 viruses during the 2013–14 and 2015–16 seasons. Recommendations for different vaccine types and specific populations are discussed. No preferential recommendation is made for one influenza vaccine product over another for persons for whom more than one licensed, recommended product is available.

Updates to the recommendations described in this report reflect discussions during public meetings of ACIP held on October 20, 2016; February 22, 2017; and June 21, 2017. New and updated information in this report includes the following:

•Vaccine viruses included in the 2017–18 U.S. trivalent influenza vaccines will be an A/Michigan/45/2015 (H1N1)pdm09–like virus, an A/Hong Kong/4801/2014 (H3N2)-like virus, and a B/Brisbane/60/2008–like virus (Victoria lineage). Quadrivalent influenza vaccines will contain these three viruses and an additional influenza B vaccine virus, a B/Phuket/3073/2013–like virus (Yamagata lineage).

• Information on recent licensures and labelling changes is discussed, including licensure of Afluria Quadrivalent (IIV4; Seqirus, Parkville, Victoria, Australia); Flublok Quadrivalent (RIV4; Protein Sciences, Meriden, Connecticut); and expansion of the age indication for FluLaval Quadrivalent (IIV4; ID Biomedical Corporation of Quebec, Quebec City, Quebec, Canada), previously licensed for ≥3 years, to ≥6 months.

• Pregnant women may receive any licensed, recommended, age-appropriate influenza vaccine.

• Afluria (IIV3; Seqirus, Parkville, Victoria, Australia) may be used for persons aged ≥5 years, consistent with Food and Drug Administration–approved labeling.

• FluMist Quadrivalent (LAIV4; MedImmune, Gaithersburg, Maryland) should not be used during the 2017–18 season due to concerns about its effectiveness against influenza A(H1N1)pdm09 viruses in the United States during the 2013–14 and 2015–16 influenza seasons.

This report focuses on the recommendations for use of vaccines for the prevention and control of influenza during the 2017–18 season in the United States. A Background Document containing further information and a summary of these recommendations are available at https://www.cdc.gov/vaccines/hcp/acip-recs/vacc-specific/flu.html. These recommendations apply to licensed influenza vaccines used within Food and Drug Administration–licensed indications, including those licensed after the publication date of this report. Updates and other information are available at CDC’s influenza website (https://www.cdc.gov/flu). Vaccination and health care providers should check CDC’s influenza website periodically for additional information.

## Introduction

Influenza viruses typically circulate widely in the United States annually, from the late fall through the early spring. Although most persons with influenza will recover without sequelae, influenza can cause serious illness and death, particularly among older adults, very young children, pregnant women, and those with certain chronic medical conditions (*1*–*6*).

Routine annual influenza vaccination for all persons aged ≥6 months who do not have contraindications has been recommended by CDC and CDC’s Advisory Committee on Immunization Practices (ACIP) since 2010 (*7*). This report updates the 2016–17 ACIP recommendations regarding the use of seasonal influenza vaccines (*8*) and provides recommendations and guidance for vaccine providers regarding the use of influenza vaccines for the 2017–18 season. A variety of different formulations of influenza vaccine are available (Table 1). Contraindications and precautions to the use of influenza vaccines are summarized (Table 2). Abbreviations are used in this report to denote the various types of vaccines (Box).

**TABLE 1:** Influenza vaccines — United States, 2017–18 influenza season*

Trade name	Manufacturer	Presentation	Age indication	Mercury (from thimerosal, *µ*g/0.5 mL)	Latex	Route
**Inactivated influenza vaccines, quadrivalent (IIV4s), standard-dose^†^**						
Afluria Quadrivalent	Seqirus	0.5 mL prefilled syringe	≥18 years	NR	No	IM^§^
		5.0 mL multidose vial	≥18 years (by needle/syringe) 18 through 64 years (by jet injector)	24.5	No	IM
Fluarix Quadrivalent	GlaxoSmithKline	0.5 mL prefilled syringe	≥3 years	NR	No	IM
FluLaval Quadrivalent	ID Biomedical Corp. of Quebec (distributed by GlaxoSmithKline)	0.5 mL prefilled syringe	≥6 months	NR	No	IM
		5.0 mL multidose vial	≥6 months	<25	No	IM
Fluzone Quadrivalent	Sanofi Pasteur	0.25 mL prefilled syringe	6 through 35 months	NR	No	IM
		0.5 mL prefilled syringe	≥3 years	NR	No	IM
		0.5 mL single-dose vial	≥3 years	NR	No	IM
		5.0 mL multidose vial	≥6 months	25	No	IM
**Inactivated influenza vaccine, quadrivalent (ccIIV4), standard-dose,^†^ cell culture-based**						
Flucelvax Quadrivalent	Seqirus	0.5 mL prefilled syringe	≥4 years	NR	No	IM
		5.0 mL multidose vial	≥4 years	25	No	IM
**Inactivated influenza vaccine, quadrivalent (IIV4), standard-dose, intradermal^¶^**						
Fluzone Intradermal Quadrivalent	Sanofi Pasteur	0.1 mL single-dose prefilled microinjection system	18 through 64 years	NR	No	ID**
**Inactivated Influenza Vaccines, trivalent (IIV3s), standard-dose^†^**						
Afluria	Seqirus	0.5 mL prefilled syringe	≥5 years	NR	No	IM
		5.0 mL multidose vial	≥5 years (by needle/syringe) 18 through 64 years (by jet injector)	24.5	No	IM
Fluvirin	Seqirus	0.5 mL prefilled syringe	≥4 years	≤1	Yes**^††^**	IM
		5.0 mL multidose vial	≥4 years	25	No	IM
**Adjuvanted inactivated influenza vaccine, trivalent (aIIV3),^†^ standard-dose**						
Fluad	Seqirus	0.5 mL prefilled syringe	≥65 years	NR	Yes**^††^**	IM
**Inactivated Influenza Vaccine, trivalent (IIV3), high-dose** ^§§^						
Fluzone High-Dose	Sanofi Pasteur	0.5 mL prefilled syringe	≥65 years	NR	No	IM
**Recombinant Influenza Vaccine, quadrivalent (RIV4)** ^¶¶^						
Flublok Quadrivalent	Protein Sciences	0.5 mL prefilled syringe	≥18 years	NR	No	IM
**Recombinant Influenza Vaccine, trivalent (RIV3)** ^¶¶^						
Flublok	Protein Sciences	0.5 mL single-dose vial	≥18 years	NR	No	IM
Live Attenuated Influenza Vaccine, quadrivalent (LAIV4)*** (not recommended for use during the 2017–18 season)						
FluMist Quadrivalent	MedImmune	0.2 mL single-dose prefilled intranasal sprayer	2 through 49 years	NR	No	NAS

**Abbreviations**: ACIP = Advisory Committee on Immunization Practices; ID = intradermal; IM = intramuscular; NAS = intranasal; NR = not relevant (does not contain thimerosal).

* Immunization providers should check Food and Drug Administration–approved prescribing information for 2017–18 influenza vaccines for the most complete and updated information, including (but not limited to) indications, contraindications, warnings, and precautions. Package inserts for U.S.-licensed vaccines are available at https://www.fda.gov/BiologicsBloodVaccines/Vaccines/ApprovedProducts/ucm093833.htm. Availability of specific products and presentations might change and differ from what is described in this table and in the text of this report.

**^†^** Standard dose intramuscular IIVs contain 15 *µ*g of each vaccine HA antigen (45 *µ*g total for trivalents and 60 *µ*g total for quadrivalents) per 0.5 mL dose.

^§^ For adults and older children, the recommended site for intramuscular influenza vaccination is the deltoid muscle. The preferred site for infants and young children is the anterolateral aspect of the thigh. Specific guidance regarding site and needle length for intramuscular administration is available in the ACIP General Best Practice Guidelines for Immunization, available at https://www.cdc.gov/vaccines/hcp/acip-recs/general-recs/index.html.

^¶^ Quadrivalent inactivated influenza vaccine, intradermal: a 0.1-mL dose contains 9 *µ*g of each vaccine HA antigen (36 *μ*g total).

** The preferred injection site is over the deltoid muscle. Fluzone Intradermal Quadrivalent is administered per manufacturer’s instructions using the delivery system included with the vaccine.

**^††^** Syringe tip cap might contain natural rubber latex.

^§§^ High-dose IIV3 contains 60 μg of each vaccine antigen (180 *μ*g total) per 0.5 mL dose.

^¶¶^ RIV contains 45 μg of each vaccine HA antigen (135 *μ*g total for trivalent 180 *μ*g total for quadrivalent) per 0.5 mL dose.

***ACIP recommends that FluMist Quadrivalent (LAIV4) not be used during the 2017–18 season.

**TABLE 2:** Contraindications and precautions to the use of influenza vaccines — United States, 2017–18 influenza season*

Vaccine type	Contraindications	Precautions
IIV	History of severe allergic reaction to any component of the vaccine^†^ or after previous dose of any influenza vaccine	Moderate-to-severe acute illness with or without fever History of Guillain-Barré syndrome within 6 weeks of receipt of influenza vaccine
RIV	History of severe allergic reaction to any component of the vaccine	Moderate-to-severe acute illness with or without fever History of Guillain-Barré syndrome within 6 weeks of receipt of influenza vaccine
LAIV For the 2017–18 season, ACIP recommends that LAIV not be used. Content is provided for information**.**	History of severe allergic reaction to any component of the vaccine^†^ or after a previous dose of any influenza vaccine Concomitant aspirin or salicylate-containing therapy in children and adolescents Children aged 2 through 4 years who have received a diagnosis of asthma or whose parents or caregivers report that a health care provider has told them during the preceding 12 months that their child had wheezing or asthma or whose medical record indicates a wheezing episode has occurred during the preceding 12 months Children and adults who are immunocompromised due to any cause (including immunosuppression caused by medications or by HIV infection) Close contacts and caregivers of severely immunosuppressed persons who require a protected environment Pregnancy Receipt of influenza antiviral medication within the previous 48 hours	Moderate-to-severe acute illness with or without fever History of Guillain-Barré syndrome within 6 weeks of receipt of influenza vaccine Asthma in persons aged ≥5 years Other underlying medical conditions that might predispose to complications after wild-type influenza infection (e.g., chronic pulmonary, cardiovascular [except isolated hypertension], renal, hepatic, neurologic, hematologic, or metabolic disorders [including diabetes mellitus])

**Abbreviations:** ACIP = Advisory Committee on Immunization Practices; IIV = Inactivated Influenza Vaccine; LAIV = Live-Attenuated Influenza Vaccine; RIV = Recombinant Influenza Vaccine.

* Immunization providers should check Food and Drug Administration–approved prescribing information for 2017–18 influenza vaccines for the most complete and updated information, including (but not limited to) indications, contraindications, and precautions. Package inserts for US-licensed vaccines are available at https://www.fda.gov/BiologicsBloodVaccines/Vaccines/ApprovedProducts/ucm093833.htm.

^†^ History of severe allergic reaction (e.g., anaphylaxis) to egg is a labeled contraindication to the use of IIV and LAIV. However, ACIP recommends that any licensed, recommended, and appropriate IIV or RIV may be administered to persons with egg allergy of any severity (see Persons with a History of Egg Allergy).

BOX **Abbreviation conventions for influenza vaccines used in this report**

- Inactivated influenza vaccines are abbreviated IIV. For the 2017–18 season, IIVs as a class will include:
  - egg-based, unadjuvanted, and adjuvanted trivalent influenza vaccines (IIV3s); and
  - egg-based or cell culture-based unadjuvanted quadrivalent influenza vaccines (IIV4s).
- RIV refers to recombinant hemagglutinin influenza vaccine, available in trivalent (RIV3) and quadrivalent (RIV4) formulations for the 2017–18 season.
- LAIV refers to live-attenuated influenza vaccine, available as a quadrivalent formulation (LAIV4) since the 2013–14 season.
- IIV, RIV, and LAIV denote vaccine categories; numeric suffix specifies the number of hemagglutinin (HA) antigens in the vaccine.
- When necessary to refer specifically to cell culture-based vaccine, the prefix “cc” is used (e.g., ccIIV4).
- When necessary to refer specifically to adjuvanted vaccine, the prefix “a” is used (e.g., aIIV3).
- When necessary to refer specifically to standard-dose or high-dose vaccines, the prefixes “SD-” or “HD-” are used (e.g., SD-IIV3 and HD-IIV3).

This report focuses on the recommendations for use of influenza vaccines for the prevention and control of influenza during the 2017–18 season in the United States. A summary of these recommendations and a Background Document containing additional information on influenza-associated illnesses and influenza vaccines are available at https://www.cdc.gov/vaccines/hcp/acip-recs/vacc-specific/flu.html.

## Methods

ACIP provides annual recommendations for the use of influenza vaccines for the prevention and control of influenza. The ACIP Influenza Work Group meets by teleconference once to twice per month throughout the year. Work Group membership includes several voting members of ACIP and representatives of ACIP Liaison Organizations.^*^ Discussions include topics such as influenza surveillance, vaccine effectiveness and safety, vaccine coverage, program feasibility, cost-effectiveness, and vaccine supply. Presentations are requested from invited experts, and published and unpublished data are discussed.

In general, the Background Document is updated to reflect recent additions to the literature related to the following: 1) recommendations that were made in previous seasons, 2) changes in the viral antigen composition of seasonal influenza vaccines, and 3) minor changes in guidance for the use of influenza vaccines (e.g., guidance for timing of vaccination and other programmatic issues, guidance for dosage in specific populations, guidance for selection of vaccines for specific populations that are already recommended for vaccination, and changes that reflect use consistent with Food and Drug Administration [FDA]–licensed indications and prescribing information). The summary included in the Background Document for such topics is not a systematic review, but is intended to provide a broad overview of current literature. In general, systematic review and evaluation of the evidence using the Grading of Recommendations, Assessment, Development and Evaluation (GRADE) approach is performed for new recommendations or substantial changes in the recommendations (e.g., expansion of the recommendation for influenza vaccination to new populations not previously recommended for vaccination or potential preferential recommendations for specific vaccines).

Updates and changes to the recommendations described in this report are of five types: 1) the vaccine virus composition for 2017–18 U.S. seasonal influenza vaccines; 2) recent regulatory actions, including new vaccine licensures and labeling changes for previously licensed vaccines; 3) updated recommendations for the use of influenza vaccines in pregnancy, including a recommendation that pregnant women may receive any licensed, recommended, age-appropriate influenza vaccine; 4) a recommendation that the trivalent inactivated influenza vaccine (IIV3) Afluria (Seqirus, Parkville, Victoria, Australia) may be used for persons aged ≥5 years, consistent with FDA-approved labeling; and 5) a recommendation (continued from the 2016–17 season) that LAIV4 not be used during the 2017–18 season. Systematic review and GRADE were not performed for these updates and changes. Information relevant to these changes includes the following:

- Recommendations for composition of Northern Hemisphere influenza vaccines are made by the World Health Organization (WHO), which organizes a consultation, generally in February of each year. Surveillance data are reviewed and candidate vaccine viruses are discussed. A summary of the WHO meeting for selection of the 2017–18 Northern Hemisphere vaccine viruses is available at http://www.who.int/influenza/vaccines/virus/recommendations/201703_recommendation.pdf. Subsequently, FDA, which has regulatory authority over vaccines in the United States, convenes a meeting of its Vaccines and Related Biological Products Advisory Committee (VRBPAC). This committee considers the recommendations of WHO, reviews and discusses similar data, and makes a final decision regarding vaccine virus composition for influenza vaccines licensed and marketed in the United States. A summary of the FDA VRBPAC meeting of March 9, 2017, at which the composition of the 2017–18 U.S. influenza vaccines was discussed, is available at https://www.fda.gov/downloads/AdvisoryCommittees/CommitteesMeetingMaterials/BloodVaccinesandOtherBiologics/VaccinesandRelatedBiologicalProductsAdvisoryCommittee/UCM552054.pdf.
- With regard to recommendations for newly licensed influenza vaccines and changes to the licensed indications for existing vaccines, ACIP relies on FDA for review of safety, immunogenicity, and effectiveness data pertaining to the licensure of influenza vaccines. Regulatory information pertinent to the two recently licensed products and one labelling change discussed in this report is available at https://www.fda.gov/BiologicsBloodVaccines/Vaccines/ApprovedProducts/ucm518291.htm (for Afluria Quadrivalent; Seqirus, Parkville, Victoria, Australia), https://www.fda.gov/BiologicsBloodVaccines/Vaccines/ApprovedProducts/ucm524660.htm (for Flublok Quadrivalent; Protein Sciences, Meriden, Connecticut), and https://www.fda.gov/BiologicsBloodVaccines/Vaccines/ApprovedProducts/ucm366061.htm (for FluLaval Quadrivalent; ID Biomedical Corporation of Quebec, Quebec City, Quebec, Canada).
- For the recommendation that pregnant women may receive any licensed, recommended, and age-appropriate influenza vaccine, because there have been no studies evaluating use of RIV3 or RIV4 in pregnancy, ACIP reviewed available information presented by the manufacturer and summarized in public documents by FDA concerning pregnancies that occurred among women participating in studies of RIV and pregnancy registry data. RIV reports to the Vaccine Adverse Event Reporting System (VAERS) also were presented. Regulatory information pertinent to this discussion is available at https://www.fda.gov/BiologicsBloodVaccines/Vaccines/ApprovedProducts/ucm524660.htm.
- For the recommendation that Afluria (IIV3; Seqirus, Parkville, Victoria, Australia) may be used for persons aged ≥5 years, ACIP reviewed data from studies performed by the manufacturer concerning the cause of an increase in the rate of febrile seizures that occurred in association with the 2010 Southern Hemisphere formulation of this product, and resulting changes in the vaccine manufacturing process. This change makes the ACIP recommendation consistent with FDA-approved labelling for this product. Minutes of the ACIP presentation of these data are available at https://www.cdc.gov/vaccines/acip/meetings/downloads/min-archive/min-2017-02.pdf. Regulatory information is available at https://www.fda.gov/BiologicsBloodVaccines/Vaccines/ApprovedProducts/ucm094043.htm.
- The recommendation that LAIV4 not be used due to concerns regarding its effectiveness against influenza A(H1N1)pdm09 viruses during the 2013–14 and 2015–16 U.S. seasons was initially made for the 2016–17 season (*9*). This recommendation continues to be made for the 2017–18 season. ACIP will continue to review data concerning LAIV4 as they become available.

## Primary Changes and Updates in the Recommendations

Routine annual influenza vaccination of all persons aged ≥6 months without contraindications continues to be recommended. No preferential recommendation is made for one influenza vaccine product over another for persons for whom more than one licensed, recommended product is available. Updated information and guidance in this report includes the following:

- 2017–18 U.S. trivalent influenza vaccines will contain an A/Michigan/45/2015 (H1N1)pdm09–like virus, an A/Hong Kong/4801/2014 (H3N2)–like virus and a B/Brisbane/60/2008–like virus (Victoria lineage). Quadrivalent vaccines will include an additional vaccine virus strain, a B/Phuket/3073/2013–like virus (Yamagata lineage). This represents a change in the influenza A(H1N1)pdm09 virus component from the previous season.
- Recent regulatory actions, including two new licensures and one labelling change are described:
  - Afluria Quadrivalent (IIV4; Seqirus, Parkville, Victoria, Australia) was licensed by FDA in August, 2016 for persons aged ≥18 years. Regulatory information is available at https://www.fda.gov/BiologicsBloodVaccines/Vaccines/ApprovedProducts/ucm518291.htm.
  - Flublok Quadrivalent (RIV4; Protein Sciences, Meriden, Connecticut) was licensed by FDA in October 2016, for persons aged ≥18 years. Regulatory information is available at https://www.fda.gov/BiologicsBloodVaccines/Vaccines/ApprovedProducts/ucm524660.htm.
  - The age indication for FluLaval Quadrivalent (IIV4; ID Biomedical Corporation of Quebec, Quebec City, Quebec, Canada) was extended from ≥3 years to ≥6 months in November 2016. Regulatory information is available at https://www.fda.gov/BiologicsBloodVaccines/Vaccines/ApprovedProducts/ucm366061.htm. Children aged 6 through 35 months may receive FluLaval Quadrivalent at the same 0.5 mL per dose (containing 15 *µ*g of hemagglutinin [HA] per vaccine virus) as is used for older children and adults. This licensure creates an additional option for vaccination of children aged 6 through 35 months, in addition to the previously available 0.25 mL per dose presentation (containing 7.5 *µ*g of HA per vaccine virus) of Fluzone Quadrivalent (IIV4; Sanofi Pasteur, Swiftwater, Pennsylvania).
- Pregnant women may receive any licensed, recommended, age-appropriate influenza vaccine.
- Afluria (IIV3; Seqirus, Parkville, Victoria, Australia) is now recommended for persons aged ≥5 years, consistent with FDA-approved labelling.
- In light of its low effectiveness against influenza A(H1N1)pdm09 in the United States during the 2013–14 and 2015–16 seasons, for the 2017–18 season, ACIP continues the recommendation that LAIV4 should not be used. Because LAIV4 is still a licensed vaccine that might be available and that some providers might elect to use, for informational purposes only, reference is made in this report to previous recommendations for its use.

## Recommendations for the Use of Influenza Vaccines, 2017–18 Season

### Groups Recommended for Vaccination

Routine annual influenza vaccination is recommended for all persons aged ≥6 months who do not have contraindications. Recommendations regarding timing of vaccination, considerations for specific populations, the use of specific vaccines, and contraindications and precautions are summarized in the sections that follow.

### Timing of Vaccination

Optimally, vaccination should occur before onset of influenza activity in the community. Health care providers should offer vaccination by the end of October, if possible. Children aged 6 months through 8 years who require 2 doses (see Children Aged 6 Months through 8 Years) should receive their first dose as soon as possible after vaccine becomes available, to allow the second dose (which must be administered ≥4 weeks later) to be received by the end of October. Although some available data indicate that early vaccination (e.g., in July and August) might be associated with suboptimal immunity before the end of the influenza season, particularly among older adults, the relative contribution of potential waning of immunity compared with those of other determinants of the impact of vaccination (e.g., timing and severity of the influenza season, the impact of missed opportunities when individuals delay vaccination and fail to return later in the season, and programmatic constraints) is unknown. Although delaying vaccination might result in greater immunity later in the season, deferral also might result in missed opportunities to vaccinate, as well as difficulties in vaccinating a population within a more constrained time period. Community vaccination programs should balance maximizing likelihood of persistence of vaccine-induced protection through the season with avoiding missed opportunities to vaccinate or vaccinating after onset of influenza circulation occurs. Revaccination later in the season of persons who have already been fully vaccinated is not recommended.

Vaccination should continue to be offered as long as influenza viruses are circulating and unexpired vaccine is available. To avoid missed opportunities for vaccination, providers should offer vaccination during routine health care visits and hospitalizations when vaccine is available. Vaccination efforts should be structured to ensure the vaccination of as many persons as possible before influenza activity in the community begins.

In any given season, the optimal time to vaccinate cannot be predicted precisely because influenza seasons vary in timing and duration. Moreover, more than one outbreak might occur in a given community in a single year. In the United States, localized outbreaks that indicate the start of seasonal influenza activity can occur as early as October. However, in 74% of influenza seasons from 1982–83 through 2015–16, peak influenza activity (which often is close to the midpoint of influenza activity for the season) has not occurred until January or later, and in 59% of seasons, the peak was in February or later (*10*).

In recent seasons, initial shipments of influenza vaccine have arrived to some vaccine providers as early as July. Very early availability of vaccine as compared with typical onset and peak of influenza activity raises questions related to the ideal time to begin vaccination. Several observational studies of influenza vaccine effectiveness have reported decreased vaccine protection within a single season, particularly against influenza A(H3N2) (*11*–*14*). In some of these studies decline in VE was particularly pronounced among older adults (*12*,*13*). Some studies have documented decline in protective antibodies over the course of one season (*15*–*17*), with antibody levels decreasing with greater time elapsed postvaccination. However, the rate and degree of decline observed has varied. Among adults in one study, HA and neuraminidase antibody levels declined slowly, with a two-fold decrease in titer estimated to take >600 days (*18*). A review of studies reporting postvaccination seroprotection rates among adults aged ≥60 years noted that seroprotection levels meeting Committee of Proprietary Medicinal Products standards were maintained for ≥4 months for the H3N2 component in all 8 studies and for the H1N1 and B components in five of seven studies (*19*). A recent multiseason analysis from the U.S. Influenza Vaccine Effectiveness (U.S. Flu VE) Network found that VE declined by about 7% per month for H3N2 and influenza B, and 6%–11% per month for H1N1pdm09 (*20*). VE remained greater than zero for at least 5 to 6 months after vaccination. Similar waning effects have not been observed consistently across age groups and virus subtypes in different populations, and the observed decline in protection could be attributable to bias, unmeasured confounding, or the late season emergence of antigenic drift variants that are less well-matched to the vaccine strain.

Vaccination efforts should continue throughout the season because the duration of the influenza season varies and influenza activity might not occur in certain communities until February or March. Providers should offer influenza vaccine routinely, and organized vaccination campaigns should continue throughout the influenza season, including after influenza activity has begun in the community. Although vaccination by the end of October is recommended, vaccine administered in December or later, even if influenza activity has already begun, is likely to be beneficial in the majority of influenza seasons.

### Guidance for Use in Specific Populations and Situations

#### Populations at Higher Risk for Medical Complications Attributable to Severe Influenza

All persons aged ≥6 months without contraindications should be vaccinated annually. However, vaccination to prevent influenza is particularly important for persons who are at increased risk for severe complications from influenza and for influenza-related outpatient, ED, or hospital visits. When vaccine supply is limited, vaccination efforts should focus on delivering vaccination to the following persons at increased risk for medical complications attributable to severe influenza who do not have contraindications (no hierarchy is implied by order of listing):

- all children aged 6 through 59 months;
- all persons aged ≥50 years;
- adults and children who have chronic pulmonary (including asthma) or cardiovascular (except isolated hypertension), renal, hepatic, neurologic, hematologic, or metabolic disorders (including diabetes mellitus);
- persons who are immunocompromised due to any cause (including immunosuppression caused by medications or by HIV infection);
- women who are or will be pregnant during the influenza season;
- children and adolescents (aged 6 months through 18 years) who are receiving aspirin- or salicylate-containing medications and who might be at risk for experiencing Reye syndrome after influenza virus infection;
- residents of nursing homes and other long-term care facilities;
- American Indians/Alaska Natives; and
- persons who are extremely obese (BMI ≥40).

ACIP recommends that LAIV4 not be used during the 2017–18 season for any population. Providers who elect to use it should consider previous guidance for use of LAIV4 for high-risk populations (Table 2).

#### Persons Who Live With or Care for Persons at Higher Risk for Influenza-Related Complications

All persons aged ≥6 months without contraindications should be vaccinated annually; however, continued emphasis should be placed on vaccination of persons who live with or care for persons at higher risk for influenza-related complications. When vaccine supply is limited, vaccination efforts should focus on delivering vaccination to persons at higher risk for influenza-related complications listed above, as well as these persons:

- health care personnel, including physicians, nurses, and other workers in inpatient and outpatient-care settings, medical emergency-response workers (e.g., paramedics and emergency medical technicians), and employees of nursing home and long-term care facilities who have contact with patients or residents, and students in these professions who will have contact with patients. ACIP guidance for immunization of health care personnel has been published previously (*21*);
- household contacts (including children) and caregivers of children aged ≤59 months (i.e., aged <5 years) and adults aged ≥50 years, particularly contacts of children aged <6 months; and
- household contacts (including children) and caregivers of persons with medical conditions that put them at high risk for severe complications from influenza.

ACIP recommends that LAIV4 not be used during the 2017–18 season for any population. Providers who elect to use it should consider previous guidance for use of LAIV4 for persons who care for or have contact with immunocompromised persons (*22*). Health care personnel and persons who are contacts of persons in these groups and who are contacts of severely immunocompromised persons (those living in a protected environment) may receive any IIV or RIV that is otherwise indicated. ACIP and HICPAC have previously recommended that health care personnel who receive LAIV should avoid providing care for severely immunosuppressed patients requiring a protected environment for 7 days after vaccination, and that hospital visitors who have received LAIV4 should avoid contact with severely immunosuppressed persons (i.e., persons requiring a protected environment) for 7 days after vaccination. However, such persons should not be restricted from visiting less severely immunosuppressed patients.

#### Children Aged 6 Months Through 8 Years

**Dose volume for children aged 6 through 35 months:** Children aged 6 through 35 months may receive one of two products at the appropriate volume for each dose needed: 0.5 mL FluLaval Quadrivalent (containing 15 *µ*g of HA per vaccine virus) or 0.25 mL Fluzone Quadrivalent (containing 7.5 *µ*g of HA per vaccine virus). These are the only two influenza vaccine products licensed for this age group. Care should be taken to administer the appropriate volume for each needed dose of either product. In either instance, the needed volume may be administered from an appropriate prefilled syringe, a single dose vial, or multidose vial, as supplied by the manufacturer. Note, however, that if a 0.5 mL single-use vial of Fluzone Quadivalent is used for a child aged 6 through 35 months, only half the volume should be administered and the other half should be discarded.

Before November 2016, the only influenza vaccine formulations licensed for children aged 6 through 35 months were the 0.25 mL (containing 7.5 *µ*g of HA per vaccine virus) dose formulations of Fluzone and Fluzone Quadrivalent. The recommendation for use of a reduced dose volume for children in this age group (half that recommended for persons aged ≥3 years) was based on increased reactogenicity noted among children (particularly younger children) following receipt of influenza vaccines in trials conducted during the 1970s. This increased reactogenicity was primarily observed with whole-virus inactivated vaccines (*23*–*27*). Studies with vaccines more similar to currently available split-virus inactivated products demonstrated less reactogenicity (*27*). Recent comparative studies of 0.5 mL vs. 0.25 mL doses of IIV3 conducted among children aged 6 through 23 months (*28*) and 6 through 35 months (*29*) noted no significant difference in reactogenicity at the higher dose. In a randomized trial comparing immunogenicity and safety of 0.5 mL FluLaval Quadrivalent with 0.25 mL Fluzone Quadrivalent, safety and reactogenicity were similar between the two vaccines. In a post-hoc analysis, superior immunogenicity was noted for the B components of FluLaval Quadrivalent among infants aged 6 through 17 months and for unprimed children (those who had not previously received at least 2 doses of influenza vaccine) aged 6 through 35 months (*30*).

**Number of doses for children aged 6 months through 8 years:** Evidence from several studies indicates that children aged 6 months through 8 years require 2 doses of influenza vaccine (administered a minimum of 4 weeks apart) during their first season of vaccination for optimal protection (*31*–*34*). Children aged 6 months through 8 years who have previously received ≥2 total doses of trivalent or quadrivalent influenza vaccine before July 1, 2017 require only 1 dose for 2017–18. The 2 doses of influenza vaccine do not have to have been administered in the same season or consecutive seasons. Children in this age group who have not previously received ≥2 doses of trivalent or quadrivalent influenza vaccine before July 1, 2017 require 2 doses for the 2017–18 season. The interval between the 2 doses should be at least 4 weeks (Figure).

**Figure:**
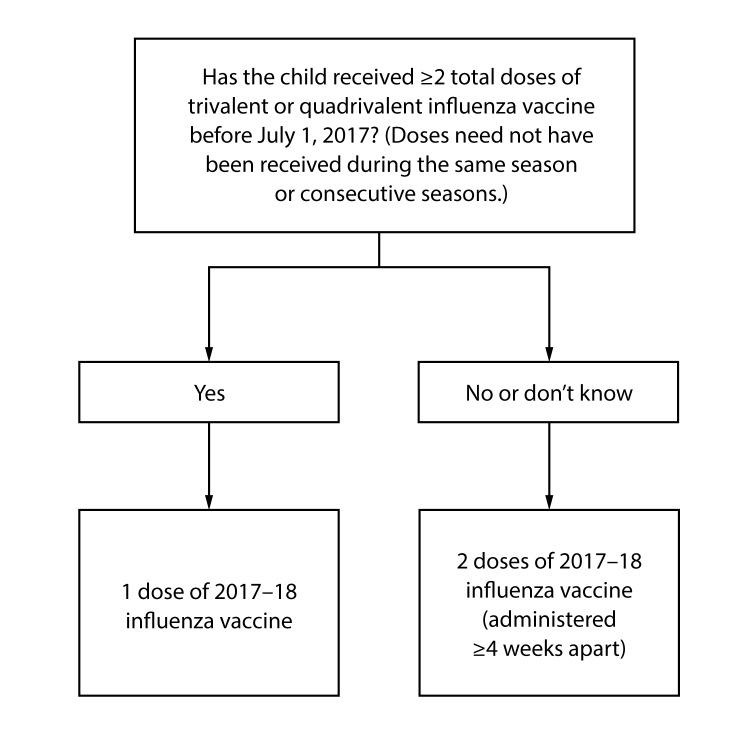
FIGURE. Influenza vaccine dosing algorithm for children aged 6 months through 8 years — Advisory Committee on Immunization Practices, United States, 2017–18 influenza season

#### Pregnant Women

Because pregnant and postpartum women are at higher risk for severe illness and complications from influenza than women who are not pregnant, ACIP recommends that all women who are pregnant or who might be pregnant in the influenza season receive influenza vaccine. Any licensed, recommended, and age-appropriate influenza vaccine may be used. Influenza vaccine can be administered at any time during pregnancy, before and during the influenza season. ACIP recommends that LAIV4 not be used in any population for the 2017–18 season. Providers should note that, as a live virus vaccine, LAIV4 should not be used during pregnancy.

Although experience with the use of IIVs is substantial, and data from observational studies are available to support the safety of these vaccines in pregnancy, data are more limited for vaccination during the first trimester (see Safety of Influenza Vaccines: Pregnant Women and Neonates in the Background Document). Moreover, there is substantially less experience with more recently licensed IIV products (e.g., quadrivalent, cell culture-based, and adjuvanted vaccines) during pregnancy in general. For RIV (available as RIV3 since the 2013–14 influenza season, and as RIV3 and RIV4 for 2017–18), data are limited to reports of pregnancies occurring incidentally during clinical trials, VAERS reports, and pregnancy registry reports. Pregnancy registries and surveillance studies exist for some products; information may be found in package inserts (*35*–*42*), available at https://www.fda.gov/BiologicsBloodVaccines/Vaccines/ApprovedProducts/ucm094045.htm for trivalent vaccines and https://www.fda.gov/BiologicsBloodVaccines/Vaccines/ApprovedProducts/ucm295057.htm for quadrivalent vaccines.

#### Older Adults

For persons aged ≥65 years, any age-appropriate IIV formulation (standard-dose or high-dose, trivalent or quadrivalent, unadjuvanted or adjuvanted) or RIV are acceptable options. Fluzone High-Dose (HD-IIV3; Sanofi Pasteur, Swiftwater, Pennsylvania) met prespecified criteria for superior efficacy to that of SD-IIV3 in a randomized trial conducted over two seasons among 31,989 persons aged ≥65 years, and might provide better protection than SD-IIV3 for this age group (*43*–*45*). In an exploratory analysis of data from a single-season randomized trial conducted among 8,604 adults aged ≥50 years, Flublok Quadrivalent (RIV4; Protein Sciences, Meriden, Connecticut) was more efficacious than SD-IIV4 (*46*,*47*); however, no claim of superiority was approved for the package insert (*47*). Fluad (aIIV3; Seqirus, Holly Springs, North Carolina) was more effective against laboratory-confirmed influenza than unadjuvanted SD-IIV3 among adults aged ≥65 years (N = 227) in an analysis from a small observational study (*48*). No preferential recommendation is made for any specific vaccine product. Vaccination should not be delayed if a specific product is not readily available.

Because of the vulnerability of this population to severe influenza illness, hospitalization, and death, efficacy and effectiveness of influenza vaccines among older adults is an area of active research (see Immunogenicity, Efficacy, and Effectiveness of Influenza Vaccines: HD-IIV3, aIIV3, and RIV4 for Older Adults in the Background Document). Recent comparative studies of efficacy/effectiveness against laboratory-confirmed influenza outcomes among older adults have focused on HD-IIV3 (Fluzone High Dose; Sanofi Pasteur, Swiftwater, Pennsylvania) (*43*,*49*–*51*), aIIV3 (Fluad, Seqirus, Holly Springs, North Carolina) (*48*), and RIV4 (Flublok Quadrivalent; Protein Sciences, Meriden, Connecticut) (*46*). Characteristics of these studies are summarized (Table 3). In each instance, the comparator vaccines have been standard dose, inactivated vaccines (SD-IIV3 as the comparator for HD-IIV3 and aIIV3; SD-IIV4 as the comparator for RIV4). No data are yet available from studies comparing the efficacy or effectiveness of HD-IIV3, aIIV3, and RIV4 with one another among older adults. This lack of comparative data prevents recommending one of these three vaccines over another for this population. HD-IIV3 exhibited superior efficacy over a comparator standard-dose IIV3 for adults aged ≥65 years in a large (N = 31,989), two-season randomized, controlled, double-blind trial (*43*,*44*), and might provide better protection than SD-IIV3s for this age group. Additional data concerning relative efficacy of HD-IIV3 for other clinical outcomes, as well as cost-effectiveness analyses and observational studies, are summarized in the Background Document. In a single-season randomized, controlled, double-blind trial comparing RIV4 with a standard-dose unadjuvanted IIV4 among adults aged ≥50 years (N = 8,604), RIV4 was more effective; however, approval for a claim of superiority was not made as a result of this exploratory analysis (*46*,*47*). Additional data, including discussion of immunogenicity studies, are described in the Background Document. Fluad (aIIV3; Seqirus, Holly Springs, North Carolina) was more effective against laboratory-confirmed influenza than unadjuvanted SD-IIV3 among adults aged ≥65 years (N = 227) in an analysis from a small observational study (*48*); no data are yet available concerning efficacy of Fluad compared with nonadjuvanted IIV3 against laboratory-confirmed influenza outcomes from a randomized trial in this population. Additional data concerning aIIV3, from studies examining immunogenicity and non-laboratory confirmed influenza outcomes, are discussed in the Background Document. ACIP will continue to review data concerning the efficacy and effectiveness of these vaccines as more information emerges.

**TABLE 3:** Summary of studies of efficacy/effectiveness of HD-IIV3, aIIV3, and RIV4 compared with unadjuvanted SD-IIVs against laboratory-confirmed influenza among older adults*

Comparator (proprietary name)	Study design	Ages enrolled	No. participants	Season(s) (predominant viruses)^†^	Location	Primary outcome	Main efficacy/effectiveness findings
**HD-IIV3 (Fluzone High-Dose, Sanofi Pasteur)**							
SD-IIV3 (Fluzone)^§^	RCT, double-blind	≥65 years	6,107 HD-IIV3 3,051 SD-IIV3	2009–10 ([H1N1]pdm09; not contained in HD-IIV3 or SD-IIV3)	U.S. (99 sites)	Culture- and/or RT-PCR-confirmed ILI, caused by types/subtypes similar to those contained in the vaccine	Primary outcome not evaluable due to emergence of (H1N1)pdm09 pandemic (no cases meeting primary endpoint of laboratory-confirmed influenza caused by viral types/subtypes similar to those in vaccine were observed; all confirmed cases were due to [H1N1]pdm09)
SD-IIV3 (Fluzone)^¶^	RCT, double-blind	≥65 years	15,990 HD-IIV3 15,993 SD-IIV3	2011–12 (H3N2) and 2012–13 ([H3N2], mismatch)	U.S., Canada (126 sites)	Laboratory confirmed (culture- and/or RT-PCR) influenza caused by any influenza viral types or subtypes, in association with protocol-defined ILI	**Culture- and/or RT-PCR-confirmed influenza; any type or subtype, with protocol-defined ILI:** All influenza: RE 24.2% (95% CI = 9.7–36.5) Influenza A: RE 24.0% (95% CI = 7.8–37.4) Influenza B: RE 25.5% (95% CI =-15.7–52.4) **Culture- and/or RT-PCR-confirmed influenza; strains similar to vaccine, with protocol-defined ILI:** All influenza: RE 35.4% (95% CI = 12.5–52.5) Influenza A: RE 31.7% (95% CI = 2.9–52.3) Influenza B: RE 45.2% (95% CI = -2.2–71.5) **Culture-confirmed influenza; any type or subtype, with protocol-defined ILI:** All influenza: RE 23.1% (95% CI = 7.5–36.2) Influenza A: RE 23.4% (95% CI = 6.0–37.6) Influenza B: RE 21.7% (95% CI =-23.8–50.8) **Culture-confirmed influenza; strains similar to vaccine. with protocol-defined ILI:** All influenza: RE 31.5% (95% CI = 4.6–51.1) Influenza A: RE 27.0% (95% CI = -8.5–51.2) Influenza B: RE 41.4% (95% CI =-10.3–69.8)
**RIV4 (Flublok Quadrivalent, Protein Sciences)**							
SD-IIV4 (Fluarix Quadrivalent)**	RCT, double-blind	≥50 years	4,303 RIV4 4,301 IIV4	2014–15 (H3N2, mismatch)	U.S. (40 sites)	RT-PCR-confirmed ILI caused by any type or subtype.	**RT-PCR-positive protocol-defined ILI;** **Aged ≥50 years:** All influenza: RE 30% (95% CI = 10–47) Influenza A: RE 36% (95%CI = 14–53) Influenza B: RE 4% (95% CI =: -72–46) **RT-PCR-positive protocol-defined ILI; Aged 50 through 64 years:** All influenza: RE 42% (95% CI = 15–61) **Aged ≥65 years:** All influenza: RE 17% (95% CI = -20–43) **Culture-positive protocol-defined ILI: Aged ≥50 years:** All influenza: RE 43% (95% CI = 21–59) Influenza A: RE 44% (95% CI = 22–61) Influenza B: RE 25% (95% CI =-121–75) **Culture-positive protocol-defined ILI:** **Aged 50–64 years:** All influenza: RE 44% (95% CI = 10–65) **Aged ≥65 years:** All influenza: RE 42% (95% CI = 9–65)
**aIIV3 (Fluad, Seqirus)**							
SD-IIV3; unvaccinated^††^	Nonrandomized, observational, prospective test negative case-control	≥65 years, presenting with symptoms of ILI	165 aIIV3 62 IIV3 55 unvaccinated	2011–12 (H3N2)	Canada (3 health authorities)	RT-PCR- confirmed ILI	**Effectiveness of aIIV3 vs. unvaccinated:** 58% (95% CI = 5–82) **Effectiveness of IIV3 vs. unvaccinated:** -2% (95% CI = -139–57) **Relative effectiveness of aIIV3 vs. IIV3:** 63% (95% CI = 4–86)

**Abbreviations:** aIIV3=trivalent inactivated influenza vaccine, adjuvanted; CI = confidence interval; ILI=influenza-like illness; HD-IIV3 = High-Dose Inactivated Influenza Vaccine, trivalent ; RE=relative efficacy (compared to active comparator vaccine); RIV4 = Recombinant Influenza Vaccine, quadrivalent; SD-IIV3 = Standard-Dose Inactivated Influenza Vaccine, trivalent.

* Studies conducted among human participants of laboratory-confirmed (by viral culture and/or RT-PCR) influenza outcomes. Modeling and medical record database studies are not included in this Table, but are discussed in the Background Document.

^†^ Information on predominant viruses is from U.S. national surveillance data (CDC, FluView, available at https://www.cdc.gov/flu/weekly/index.htm).

^§^ **Source:** DiazGranados CA, Dunning AJ, Jordanov E, Landolfi V, Denis M, Talbot HK. High-dose trivalent influenza vaccine compared to standard dose vaccine in elderly adults: safety, immunogenicity and relative efficacy during the 2009–2010 season. Vaccine 2013;31:861–6.

^¶^ **Source:** DiazGranados CA, Dunning AJ, Kimmel M, et al. Efficacy of high-dose versus standard-dose influenza vaccine in older adults. N Engl J Med 2014;371:635–45.

** **Source:** Dunkle LM, Izikson R, Patriarca P, et al.; PSC12 Study Team. Efficacy of recombinant influenza vaccine in adults 50 years of age or older. N Engl J Med 2017;376:2427–36.

^††^ **Source:** Van Buynder PG, Konrad S, Van Buynder JL, et al. The comparative effectiveness of adjuvanted and unadjuvanted trivalent inactivated influenza vaccine (TIV) in the elderly. Vaccine 2013;31:6122–8.

#### Immunocompromised Persons

Immunocompromised states comprise a heterogeneous range of conditions. In many instances, limited data are available regarding the use of influenza vaccines in the setting of specific immunocompromised states. ACIP recommends that LAIV4 not be used in any population for the 2017–18 season; providers considering its use should note that live virus vaccines should not be used for persons with most forms of altered immunocompetence (*52*), given the uncertain but biologically plausible risk for disease attributable to the vaccine virus. In addition to potential safety issues, immune response to live or inactivated vaccines might be blunted in some clinical situations, such as for persons with congenital immune deficiencies, persons receiving cancer chemotherapy, and persons receiving immunosuppressive medications. For this reason, timing of vaccination might be a consideration (e.g., vaccinating during some period either before or after an immunocompromising intervention).

The Infectious Diseases Society of America (IDSA) has published detailed guidance for the selection and timing of vaccines for persons with specific immunocompromising conditions, including congenital immune disorders, stem cell and solid organ transplant, anatomic and functional asplenia, and therapeutic drug-induced immunosuppression, as well as for persons with cochlear implants or other conditions leading to persistent cerebrospinal fluid-oropharyngeal communication (*53*). ACIP will continue to review accumulating data on use of influenza vaccines in these contexts.

#### Persons with a History of Guillain-Barré Syndrome Following Influenza Vaccination

A history of Guillain-Barré Syndrome (GBS) within 6 weeks following a previous dose of any type of influenza vaccine is considered a precaution to vaccination (Table 2). Persons who are not at high risk for severe influenza complications (see Populations at Higher Risk for Medical Complications Attributable to Severe Influenza) and who are known to have experienced GBS within 6 weeks of a previous influenza vaccination generally should not be vaccinated. As an alternative to vaccination, physicians might consider using influenza antiviral chemoprophylaxis for these persons (*54*). However, the benefits of influenza vaccination might outweigh the risks for certain persons who have a history of GBS and who also are at high risk for severe complications from influenza.

#### Persons with a History of Egg Allergy

As is the case for other vaccines, influenza vaccines contain various different components that might cause allergic and anaphylactic reactions. Not all such reactions are related to egg proteins; however, the possibility of reactions to influenza vaccines in egg-allergic persons might be of concern to these persons and vaccine providers. Currently available influenza vaccines, with the exceptions of RIV3, RIV4 and ccIIV4, are prepared by propagation of virus in embryonated eggs. Only RIV3 and RIV4 are considered egg-free. For ccIIV4 (Flucelvax Quadrivalent; Seqirus, Holly Springs, North Carolina), ovalbumin is not directly measured. During manufacture of ccIIV4, viruses are propagated in mammalian cells rather than in eggs; however, some of the viruses provided to the manufacturer are egg-derived, and therefore egg proteins may potentially be introduced at the start of the manufacturing process. Once these viruses are received by the manufacturer, no eggs are used, and dilutions at various steps during the manufacturing process result in a theoretical maximum of 5x10^-8^ *μ*g/0.5 mL dose of total egg protein (Seqirus, unpublished data, 2016).

Severe allergic reactions to vaccines, although rare, can occur at any time, despite a recipient’s allergy history. Therefore, all vaccine providers should be familiar with the office emergency plan, and be certified in cardiopulmonary resuscitation (*52*). For persons who report a history of egg allergy, ACIP recommends the following (based upon the recipient’s previous symptoms after exposure to egg):

- Persons with a history of egg allergy who have experienced only urticaria (hives) after exposure to egg should receive influenza vaccine. Any licensed and recommended influenza vaccine (i.e., any IIV or RIV) that is otherwise appropriate for the recipient’s age and health status may be used.
- Persons who report having had reactions to egg involving symptoms other than urticaria (hives), such as angioedema, respiratory distress, lightheadedness, or recurrent emesis; or who required epinephrine or another emergency medical intervention, may similarly receive any licensed and recommended influenza vaccine (i.e., any IIV or RIV) that is otherwise appropriate for the recipient’s age and health status. The selected vaccine should be administered in an inpatient or outpatient medical setting (including, but not necessarily limited to, hospitals, clinics, health departments, and physician offices). Vaccine administration should be supervised by a health care provider who is able to recognize and manage severe allergic conditions.
- A previous severe allergic reaction to influenza vaccine, regardless of the component suspected of being responsible for the reaction, is a contraindication to future receipt of the vaccine.

No period of postvaccination observation period is recommended specifically for egg-allergic persons. However, ACIP recommends that vaccine providers consider observing patients for 15 minutes following administration of any vaccine to decrease the risk for injury should syncope occur (*52*).

Persons who are able to eat lightly cooked egg (e.g., scrambled egg) without reaction are unlikely to be allergic. Egg-allergic persons might tolerate egg in baked products (e.g., bread or cake). Tolerance to egg-containing foods does not exclude the possibility of egg allergy. Egg allergy can be confirmed by a consistent medical history of adverse reactions to eggs and egg-containing foods, plus skin and/or blood testing for immunoglobulin E directed against egg proteins (*55*).

Occasional cases of anaphylaxis in egg-allergic persons have been reported to VAERS after administration of influenza vaccines (*56*,*57*). ACIP will continue to review available data regarding anaphylaxis cases following influenza vaccines.

#### Vaccination Issues for Travelers

Travelers who want to reduce the risk for influenza infection should consider influenza vaccination, preferably at least 2 weeks before departure. In particular, persons residing in the United States who are at high risk for complications of influenza and who were not vaccinated with influenza vaccine during the preceding Northern Hemisphere fall or winter should consider receiving influenza vaccine before departure if they plan to travel:

- to the tropics,
- with organized tourist groups or on cruise ships, or
- to the Southern Hemisphere during the Southern Hemisphere influenza season (April–September).

No information is available indicating a benefit to revaccinating persons before summer travel who already were vaccinated during the preceding fall. In many cases, revaccination will not be feasible as Southern Hemisphere formulations of influenza vaccine are not generally available in the United States. Persons at high risk who receive the previous season’s vaccine before travel should receive the current vaccine the following fall or winter. Persons at higher risk for influenza complications should consult with their health care practitioner to discuss the risk for influenza or other travel-related diseases before embarking on travel during the summer.

In temperate climate regions of the Northern and Southern hemispheres, influenza activity is seasonal, occurring approximately from October through May in the Northern Hemisphere and April through September in the Southern Hemisphere. In the tropics, influenza occurs throughout the year. Travelers can be exposed to influenza when travelling to an area where influenza is circulating, or when traveling as part of large tourist groups (e.g., on cruise ships) that include persons from areas of the world in which influenza viruses are circulating (*58*,*59*). In a survey of Swiss travelers to tropical and subtropical countries, among 211 who reported febrile illness during or after travelling abroad and who provided paired serum samples, 40 demonstrated serologic evidence of influenza infection (*60*). Among 109 travelers returning to Australia from travel in Asia who reported acute respiratory infection symptoms, four (3.7%) had evidence of influenza A infection (evidenced by fourfold rise in antibody titer) (*61*).

Influenza vaccine formulated for the Southern Hemisphere might differ in viral composition from the Northern Hemisphere vaccine. However, with the exception of the Southern Hemisphere formulation of Fluzone Quadrivalent (IIV4; Sanofi Pasteur, Swiftwater, Pennsylvania), Southern Hemisphere formulation seasonal influenza vaccines are not licensed in the United States, and Southern Hemisphere formulations generally are not commercially available in the United States. More information on influenza vaccines and travel is available at https://www.cdc.gov/flu/travelers/travelersfacts.htm***.***

#### Use of Influenza Antiviral Medications

Administration of IIV or RIV to persons receiving influenza antiviral medications for treatment or chemoprophylaxis is acceptable. ACIP recommends that LAIV4 should not be used during the 2017–18 season. If used, providers should note that influenza antiviral medications may reduce the effectiveness of LAIV4 if given within 48 hours before to 14 days after LAIV4 (*62*). Persons who receive influenza antiviral medications during this period surrounding receipt of LAIV4 may be revaccinated with another appropriate vaccine formulation (e.g., IIV or RIV).

#### Concurrent Administration of Influenza Vaccine with Other Vaccines

Data regarding potential interference following simultaneous or sequential administration for the many potential combinations of vaccines are limited. Therefore, following the ACIP General Best Practice Guidelines for Immunization is prudent (*52*). IIVs and RIV may be administered concurrently or sequentially with other inactivated vaccines or with live vaccines. LAIV4 is not recommended for use in 2017–18. Providers considering its use should note that although inactivated or live vaccines can be administered simultaneously with LAIV4, after administration of a live vaccine (such as LAIV4), at least 4 weeks should pass before another live vaccine is administered.

Relatively limited data are available on the concurrent administration of influenza vaccines with other vaccines. In a study comparing the immunogenicity of IIV and zoster vaccine given either concurrently or separated by a 4-week interval to adults aged ≥50 years, antibody responses were similar for both schedules (*63*). In some studies, reduced responses have been noted to PCV13 (*64*,*65*), tetanus antigens (*66*), and pertussis antigens (*66*) when co-administered with IIV; in most instances the clinical significance of this is uncertain. Reassuring safety profiles have been noted for simultaneous administration of zoster vaccine (*63*), PCV13 (*64*,*65*), PPSV23 (*67*) and Tdap (*66*) among adults and of Tdap among pregnant women (*68*). Increased prevalence of local and/or systemic adverse reactions have been noted with concurrent administration in some of these studies, but these symptoms have generally been reported to be mild or moderate. Among children, co-administration of IIV and PCV13 was associated with increased risk of fever on the day of vaccination and the day following (i.e., days 0–1 postvaccination) after vaccination in children aged 6 through 23 months in a study conducted during the 2011–12 season (*69*). Increased risk of febrile seizure in this age group has been noted within days 0–1 following coadministration of IIV with PCV7, PCV13, or DTaP-containing vaccines during the 2006–07 through 2010–11 seasons (*70*), and with PCV13 during the 2014–15 season (*71*). No changes in the recommendations for administration of these vaccines were made, and these vaccines may be given concomitantly. Surveillance of febrile seizures is ongoing through VAERS, and the Vaccine Safety Datalink annual influenza vaccine safety surveillance includes monitoring for seizures following vaccination.

Concurrent administration to children of LAIV3 with MMR and varicella vaccine was not associated with diminished immunogenicity to antigens in any of the vaccines in one study (*72*); diminished response to rubella was observed in another examining coadministraion of LAIV3 and MMR (*73*). Administration of OPV was not associated with interference when administered with LAIV (*74*). No safety concerns were revealed in these studies.

## Influenza Vaccine Composition and Available Products

### Influenza Vaccine Composition for the 2017–18 Season

All influenza vaccines licensed in the United States will contain components derived from influenza viruses antigenically similar to those recommended by FDA (*75*). Both trivalent and quadrivalent influenza vaccines will be available in the United States. The 2017–18 U.S. influenza vaccines will contain the following components:

- an A/Michigan/45/2015 (H1N1)pdm09–like virus,
- an A/Hong Kong/4801/2014 (H3N2)–like virus, and
- a B/Brisbane/60/2008–like virus (Victoria lineage).

The 2017–18 U.S. quadrivalent vaccines will contain the same three antigens and an additional influenza B virus component, a B/Phuket/3073/2013–like virus (Yamagata lineage). Compared with 2016–17, the composition for 2017–18 represents a change in the influenza A(H1N1)pdm09–like virus.

### Vaccine Products for the 2017–18 Season

A variety of influenza vaccine products are licensed and available from several different manufacturers (Table 1). For many vaccine recipients, more than one type or brand of vaccine might be appropriate within approved indications and ACIP recommendations. A licensed, age-appropriate influenza vaccine product should be used. Not all products are likely to be uniformly available in any practice setting or locality. Vaccination should not be delayed in order to obtain a specific product when an appropriate one is already available. Within these guidelines and approved indications, where more than one type of vaccine is appropriate and available, no preferential recommendation is made for use of any influenza vaccine product over another.

Since the publication of the previous season’s guidelines, two new influenza vaccine products have been licensed; Afluria Quadrivalent (IIV4; Seqirus, Parkville, Victoria, Australia) and Flublok Quadrivalent (RIV4; Protein Sciences, Meriden, Connecticut). In addition, a labeling change has been approved for a previously-licensed product: FluLaval Quadrivalent (IIV4, ID Biomedical Corporation of Quebec, Quebec City, Quebec, Canada) is now licensed for children aged 6 months and older. These are described in the New Influenza Vaccine Product Approvals section. New licensures and changes to FDA-approved labeling might occur subsequent to this report. These recommendations apply to all licensed influenza vaccines used within FDA–licensed indications, including changes in FDA-approved labeling that might occur after publication of this report. As these changes occur, they will be reflected in the online version of Table 1, available at https://www.cdc.gov/flu/protect/vaccine/vaccines.htm.

### Dosage, Administration, Contraindications, and Precautions

#### Inactivated Influenza Vaccines (IIVs)

**Available products:** IIVs comprise multiple products (Table 1). Both quadrivalent and trivalent formulations are available.

With one exception, U.S.-licensed IIVs are manufactured through propagation of virus in eggs. The exception, the cell culture-based vaccine Flucelvax Quadrivalent (ccIIV4; Seqirus, Holly Springs, North Carolina), contains vaccine viruses propagated in Madin-Darby canine kidney cells. Flucelvax Quadrivalent is not considered egg-free, as some of the initial vaccine viruses provided to the manufacturer by WHO are egg-derived. For the 2017–18 season, the influenza A (H1N1) and both influenza B components will be egg-derived; the influenza A (H3N2) component will be cell-derived.

With one exception, IIVs licensed in the United States contain no adjuvant. The exception, Fluad (aIIV3; Seqirus, Holly Springs, North Carolina) contains the adjuvant MF59.

There are IIVs that are licensed for persons as young as age 6 months. However, age indications for the various individual IIVs differ (Table 1). Only age-appropriate products should be administered. Afluria (IIV3, Seqirus, Parkville, Victoria, Australia), which was previously recommended for persons aged ≥9 years, is now recommended for persons aged ≥5 years. Providers should consult package inserts and updated CDC/ACIP guidance for current information.

**Dosage and administration:** All IIV preparations contain 15 *µ*g of HA per vaccine virus strain (45 *µ*g total for IIV3s and 60 *µ*g total for IIV4s) per 0.5 mL dose, with two exceptions. Fluzone High Dose (HD-IIV3; Sanofi Pasteur, Swiftwater, Pennsylvania), an IIV3 licensed for persons aged ≥65 years, contains 60 *µ*g of each HA per vaccine virus strain (180 *µ*g total) (*44*). Fluzone Intradermal Quadrivalent (intradermal IIV4; Sanofi Pasteur, Swiftwater, Pennsylvania), an intradermally administered IIV4 licensed for persons aged 18 through 64 years, contains 9 *µ*g of each HA per vaccine virus strain (36 *µ*g total) (*36*).

For children aged 6 through 35 months, two IIV products are licensed by FDA. The approved dose volumes differ for these two products. Children in this age group may receive either 1) 0.5 mL of FluLaval Quadrivalent (ID Biomedical Corporation of Quebec, Quebec City, Quebec, Canada) (*37*), which contains 15 *µ*g of HA per virus, or 2) 0.25 mL dose Fluzone Quadrivalent (Sanofi Pasteur, Swiftwater, Pennsylvania) (*35*), which contains 7.5 *µ*g of HA per virus. Care must be taken to administer each at the appropriate dose for each product in this age group. If prefilled syringes are not available, the appropriate dose can be taken from a single-use or multidose vial, at the appropriate volume for the given product.

Children aged 36 months through 17 years (for whom only intramuscular IIVs are licensed) and adults aged ≥18 years who are receiving intramuscular preparations of IIV should receive 0.5 mL per dose. If a smaller intramuscular vaccine dose (e.g., 0.25 mL) is administered inadvertently to an adult, an additional 0.25 mL dose should be administered to provide a full dose (0.5 mL). If the error is discovered later (after the patient has left the vaccination setting), a full 0.5 mL dose should be administered as soon as the patient can return. Vaccination with a formulation approved for adult use should be counted as a dose if inadvertently administered to a child.

With the exception of Fluzone Intradermal Quadrivalent (Sanofi Pasteur, Swiftwater, Pennsylvania), IIVs are administered intramuscularly. For adults and older children, the deltoid is the preferred site. Infants and younger children should be vaccinated in the anterolateral thigh. Additional specific guidance regarding site selection and needle length for intramuscular administration are provided in the ACIP General Best Practice Guidelines for Immunization (*52*). Fluzone Intradermal Quadrivalent is administered intradermally, preferably over the deltoid muscle, using the included delivery system (*36*). Two IIVs, Afluria and Afluria Quadrivalent (Seqirus, Parkville, Victoria, Australia), are licensed for intramuscular administration via jet injector (Stratis; Pharmajet, Golden, Colorado) for persons aged 18 through 64 years (*39*,*76*).

**Trivalent versus quadrivalent IIVs:** Both trivalent and quadrivalent IIVs will be available during the 2017–18 season. Quadrivalent vaccines contain one virus from each of the two influenza B lineages (one B/Victoria virus and one B/Yamagata virus), whereas trivalent vaccines contain one influenza B virus from one lineage. Quadrivalent vaccines are thus designed to provide broader protection against circulating influenza B viruses. However, no preference is expressed for either IIV3 or IIV4.

**Contraindications and precautions for the use of IIVs:** Manufacturer package inserts and updated CDC/ACIP guidance should be consulted for current information on contraindications and precautions for individual vaccine products. In general, history of severe allergic reaction to the vaccine or any of its components (including egg) is a labeled contraindication to the receipt of IIVs (Table 2). However, ACIP makes specific recommendations for the use of influenza vaccine for persons with egg allergy (see Persons with a History of Egg Allergy). Influenza vaccine is not recommended for persons with a history of severe allergic reaction to the vaccine or to components other than egg. Information about vaccine components is located in package inserts from each manufacturer. Prophylactic use of antiviral agents is an option for preventing influenza among persons who cannot receive vaccine (*54*).

Moderate or severe acute illness with or without fever is a general precaution for vaccination (*52*). GBS within 6 weeks following a previous dose of influenza vaccine is considered a precaution for use of influenza vaccines (Table 2).

#### Recombinant Influenza Vaccine (RIVs)

**Available products:** Two RIV products, Flublok (RIV3) and Flublok Quadrivalent (RIV4), are expected to be available for the 2017–18 influenza season. RIV3 and RIV4 are indicated for persons aged ≥18 years. RIVs are manufactured without the use of influenza viruses; therefore, similarly to IIVs, no shedding of vaccine virus will occur. These vaccines are produced without the use of eggs, and are egg-free. No preference is expressed for RIVs versus IIVs within specified indications.

**Dosage and administration:** RIVs are administered by intramuscular injection. A 0.5 mL dose contains 45 *µ*g of HA derived from each vaccine virus (135 *µ*g total for RIV3 and 180 *µ*g total for RIV4).

**Trivalent versus quadrivalent RIV:** Both trivalent and quadrivalent RIV will be available during the 2017–18 season. Quadrivalent vaccines contain one virus from each of the two influenza B lineages (one B/Victoria virus and one B/Yamagata virus), whereas trivalent vaccines contain one influenza B virus from one lineage. Quadrivalent vaccines are thus designed to provide broader protection against circulating influenza B viruses. However, no preference is expressed for either RIV3 or RIV4.

**Contraindications and precautions for use of RIV:** RIVs are contraindicated in persons who have had a severe allergic reaction to any component of the vaccine. Moderate or severe acute illness with or without fever is a general precaution for vaccination (*52*). GBS within 6 weeks following a previous dose of influenza vaccine is considered a precaution for use of influenza vaccines (Table 2). Flublok is not licensed for use in children aged <18 years.

#### Live Attenuated Influenza Vaccine (LAIV4)

Pending further data, for the 2017–18 season, ACIP recommends that LAIV4 not be used because of concerns regarding its effectiveness against influenza A(H1N1)pdm09 viruses in the United States during the 2013–14 and 2015–16 seasons. As it is a licensed vaccine and might be available during 2017–18, the material in this section is provided for information.

**Dosage and administration:** LAIV4 is administered intranasally using the supplied prefilled, single-use sprayer containing 0.2 mL of vaccine. Approximately 0.1 mL (i.e., half of the total sprayer contents) is sprayed into the first nostril while the recipient is in the upright position. An attached dose-divider clip is removed from the sprayer to administer the second half of the dose into the other nostril. If the vaccine recipient sneezes immediately after administration, the dose should not be repeated. However, if nasal congestion is present that might impede delivery of the vaccine to the nasopharyngeal mucosa, deferral of administration should be considered until resolution of the illness, or another appropriate vaccine should be administered instead.

**Contraindications and precautions:** ACIP recommends that LAIV4 not be used during the 2017–18 season. Previously issued guidance regarding contraindications and precautions is provided for informational purposes only (Table 2).

### New Influenza Vaccine Product Approvals

Since the publication of the previous season’s guidance, there have been two new product approvals (Afluria Quadrivalent and Flublok Quadrivalent) and one change to the approved indication for an existing product (expansion of the age indication for FluLaval Quadrivalent from ≥3 years to ≥6 months).

#### Afluria Quadrivalent (IIV4)

Afluria Quadrivalent (IIV4, Seqirus, Parkville, Victoria, Australia) was licensed by FDA in August 2016, for persons aged ≥18 years, and was available during the 2016–17 season alongside the trivalent formulation of Afluria. In a prelicensure study of the safety and immunogenicity of Afluria Quadrivalent compared with two formulations of Afluria (each containing one of the two influenza B viruses contained in the quadrivalent) among persons aged ≥18 years, Afluria Quadrivalent met pre-specified criteria for immunologic noninferiority for all four vaccine viruses, and criteria for immunologic superiority for each B virus as compared to the trivalent formulation containing the alternate B virus. 

Some local injection site reactions were more common among those who received Afluria Quadrivalent, including an imbalance of Grade 3 injection site induration/swelling at 0.3% in the Afluria Quadrivalent group and 0.06% in the pooled Afluria trivalent groups), but rates of these reactions were low overall (*77*,*78*).

#### Flublok Quadrivalent (RIV4)

Flublok Quadrivalent (RIV4; Protein Sciences, Meriden, Connecticut) was licensed by FDA in October 2016, for persons aged ≥18 years. It is anticipated that Flublok Quadrivalent will be available for the 2017–18 season, alongside the trivalent formulation of Flublok. In a prelicensure analysis of immunogenicity data from a subset of participants enrolled in a randomized relative efficacy trial comparing Flublok Quadrivalent with a licensed comparator standard-dose IIV4 among persons aged ≥50 years during the 2014–15 season, Flublok Quadrivalent met criteria for noninferiority to the comparator IIV4 for the A(H3N2) and B/Yamagata antigens, but not for the A(H1N1) or B/Victoria antigens (*47*). In an exploratory analysis of data from this trial (N = 8,604), Flublok Quadrivalent demonstrated 30% greater relative efficacy (95% confidence interval [CI] = 10–47) over IIV4 (*46*,*47*). In a second prelicensure study, evaluating safety, reactogenicity, and immunogenicity compared with a licensed IIV4 among persons aged 18 through 49 years during the 2014–15 season, Flublok Quadrivalent met criteria for noninferiority to the comparator IIV4 for the A(H1N1), A(H3N2) and B/Yamagata antigens, but not for the B/Victoria antigen. Safety data from both studies suggested comparable safety to the comparator IIV4 for persons aged ≥18 years (*47*).

#### FluLaval Quadrivalent (IIV4)

In November 2016, FDA approved expansion of the licensed age indication for FluLaval Quadrivalent (IIV4; ID Biomedical Corporation of Quebec, Quebec City, Quebec, Canada). Previously licensed for persons aged ≥3 years, FluLaval Quadrivalent is now licensed for persons aged ≥6 months. The approved dose volume is 0.5 mL for all ages. This represents a new option for vaccination of children aged 6 through 35 months, for whom previously the only approved influenza vaccine formulation was the 0.25 mL dose volume of Fluzone Quadrivalent. With this approval, children in this age group may receive either 0.5 mL of FluLaval Quadrivalent or 0.25 mL of Fluzone Quadrivalent for each dose needed.

In a prelicensure study comparing the immunogenicity and safety of 0.5 mL of FluLaval Quadrivalent to that of 0.25 mL of Fluzone Quadrivalent among children aged 6 through 35 months, FluLaval Quadrivalent met criteria for immunogenic noninferiority for all four vaccine strains. Safety and reactogenicity were similar between the two vaccines (*30*,*79*).

### Storage and Handling of Influenza Vaccines

In all instances, approved manufacturer packaging information should be consulted for authoritative guidance concerning storage and handling of all influenza vaccines. Vaccines should be protected from light and stored at recommended temperatures. In general, influenza vaccines are recommended to be stored refrigerated between 2° to 8°C (36° to 46°F) and should not be frozen. Vaccine that has frozen should be discarded. In addition, the cold chain must be maintained when LAIV4 is transported. Single-dose vials should not be accessed for more than one dose. Multiple-dose vials should be returned to recommended storage conditions between uses, and once first accessed should not be kept beyond the recommended period of time. For information on permissible temperature excursions and other departures from recommended storage conditions that are not discussed in the package labelling, contact the manufacturer. Vaccines should not be used after the expiration date on the label.

## Additional Sources for Information Regarding Influenza and Vaccines

### Influenza Surveillance, Prevention, and Control

Updated information regarding influenza surveillance, detection, prevention, and control is available at https://www.cdc.gov/flu. U.S surveillance data are updated weekly during October–May on FluView (https://www.cdc.gov/flu/weekly). In addition, periodic updates regarding influenza are published in *MMWR* (https://www.cdc.gov/mmwr). Additional information regarding influenza vaccine can be obtained from CDC by calling 1-800-232-4636. State and local health departments should be consulted about availability of influenza vaccine, access to vaccination programs, information related to state or local influenza activity, reporting of influenza outbreaks and influenza-related pediatric deaths, and advice concerning outbreak control.

### Vaccine Adverse Event Reporting System

The National Childhood Vaccine Injury Act of 1986 requires health care providers to report any adverse event listed by the vaccine manufacturer as a contraindication to further doses of the vaccine, or any adverse event listed in the VAERS Table of Reportable Events Following Vaccination (https://vaers.hhs.gov/docs/VAERS_Table_of_Reportable_Events_Following_Vaccination.pdf) that occurs within the specified time period after vaccination. In addition to mandated reporting, health care providers are encouraged to report any clinically significant adverse event following vaccination to VAERS. Information on how to report a vaccine adverse event is available at https://vaers.hhs.gov/index.html. Additional information on VAERS and vaccine safety is available by emailing info@vaers.org or by calling 1-800-822-7967.

### National Vaccine Injury Compensation Program

The National Vaccine Injury Compensation Program (VICP), established by the National Childhood Vaccine Injury Act of 1986, as amended, provides a mechanism through which compensation can be paid on behalf of a person determined to have been injured or to have died as a result of receiving a vaccine covered by VICP. The Vaccine Injury Table (https://www.hrsa.gov/vaccinecompensation/vaccineinjurytable.pdf) lists the vaccines covered by VICP and the associated injuries and conditions (including death) that might receive a legal presumption of causation. If the injury or condition is not on the Table, or does not occur within the specified time period on the Table, persons must prove that the vaccine caused the injury or condition. Eligibility for compensation is not affected by whether a covered vaccine is used off-label or inconsistently with recommendations.

To be eligible for compensation under VICP, a claim must be filed within 3 years after the first symptom of the vaccine injury. Death claims must be filed within 2 years of the vaccine-related death and not more than 4 years after the start of the first symptom of the vaccine-related injury from which the death occurred. When a new vaccine is covered by VICP or when a new injury/condition is added to the Table, claims that do not meet the general filing guidelines must be filed within 2 years from the date the vaccine or injury/condition is added to the Table for injuries or deaths that occurred up to 8 years before the Table change (*80*). Persons of all ages who receive a VICP-covered vaccine might be eligible to file a claim. Additional information is available at https://www.hrsa.gov/vaccinecompensation or by calling 1-800-338-2382.

### Additional Resources

#### ACIP Statements

- General Best Practice Guidelines for Immunization: Best Practices Guidance of the Advisory Committee on Immunization Practices (ACIP). https://www.cdc.gov/vaccines/hcp/acip-recs/general-recs/index.html
- Immunization of Healthcare Personnel: Recommendations of the Advisory Committee on Immunization Practices (ACIP), 2011. MMWR Recomm Rep 2011;60(No. RR-7). https://www.cdc.gov/mmwr/preview/mmwrhtml/rr6007a1.htm
- Recommended Immunization Schedules for Adults, 2017: https://www.cdc.gov/vaccines/schedules/hcp/adult.html
- Recommended Immunization Schedule for Children and Adolescents Aged 18 years or Younger, 2017: https://www.cdc.gov/vaccines/schedules/hcp/child-adolescent.html

#### Vaccine Information Sheets (VISs)

- VIS for IIV and RIV: https://www.cdc.gov/vaccines/hcp/vis/vis-statements/flu.pdf
- VIS for LAIV: https://www.cdc.gov/vaccines/hcp/vis/vis-statements/flulive.pdf

#### Influenza Vaccine Package Inserts

- Trivalent Vaccines: https://www.fda.gov/BiologicsBloodVaccines/Vaccines/ApprovedProducts/ucm094045.htm
- Quadrivalent Vaccines: https://www.fda.gov/BiologicsBloodVaccines/Vaccines/ApprovedProducts/ucm295057.htm

#### CDC Influenza Antiviral Guidance

- Antiviral Drugs: Information for Healthcare Professionals: https://www.cdc.gov/flu/professionals/antivirals/index.htm

#### American Academy of Pediatrics (AAP) Guidance

- AAP Recommendations for Prevention and Control of Influenza in Children: https://redbook.solutions.aap.org/ss/influenza-resources.aspx

#### Infectious Diseases Society of America (IDSA) Guidance

- 2013 IDSA Clinical Practice Guideline for Vaccination of the Immunocompromised Host: https://academic.oup.com/cid/article/58/3/e44/336537/2013–IDSA-Clinical-Practice-Guideline-for*A list of Work Group members may be found on page 20 of this report.
